# Mesenchymal Stromal Cells in Pediatric Hematopoietic Cell Transplantation a Review and a Pilot Study in Children Treated With Decidua Stromal Cells for Acute Graft-versus-Host Disease

**DOI:** 10.3389/fimmu.2020.567210

**Published:** 2020-10-19

**Authors:** Olle Ringdén, Britt Gustafsson, Behnam Sadeghi

**Affiliations:** ^1^Translational Cell Therapy Research (TCR), Division of Pediatrics, Department of Clinical Science, Intervention and Technology (CLINTEC), Karolinska Institutet, Stockholm, Sweden; ^2^Division of Pediatrics, CLINTEC, Karolinska Institutet, Stockholm, Sweden

**Keywords:** graft-versus-host disease (GVHD), mesenchymal stromal cell (MSC), pediatric haematopoietic stem cell transplantation, cell theraphy, decidua stromal cells (DSCs)

## Abstract

Mesenchymal stromal cells (MSCs) are rare precursors in all organs of the body. MSCs have profound anti-inflammatory effects and reduce alloreactivity *in vitro* and *in vivo*. In pediatric allogeneic hematopoietic cell transplantation (HCT), MSCs have mainly been used to treat acute graft-versus-host disease (GVHD). MSCs are commercially available for this indication in Canada, Japan, and New Zeeland. More rare indications for MSCs in pediatric patients include graft failure and chronic GVHD. MSCs from bone marrow, adipose tissue, umbilical cord, Wharton's jelly, placenta tissue, and decidua have been used, but the optimal clinical stromal cell source has not been compared in clinical trials. More experimental clinical indications using MSCs, such as sepsis, acute respiratory distress syndrome, hemorrhages, pneumo-mediastinum, and neuroinflammation have primarily been explored in animal models or adult HCT patients. MSCs have almost no if any side-effects. In this pilot study we report the outcome of six children treated with decidua stromal cells (DSCs) for steroid refractory acute GVHD. At 6 months, complete response was seen in four patients and partial response in two patients. One child with high-risk ALL died from relapse and a boy with sickle cell disease died from a cerebral hemorrhage. Five-year survival was 67% and all survivors showed a Lansky score of 100%. To conclude, MSCs from various organs are well-tolerated and have shown an encouraging outcome for acute GVHD in pediatric patients.

## Introduction

Hematopoietic cell transplantation (HCT) is an established treatment for children with both malignant and non-malignant hematopoietic diseases and inborn errors of metabolism (1–4). The main obstacles to success are relapse of the disease, infections, graft failure, toxicity of various organs, hemorrhagic cystitis, and graft-versus-host disease (GVHD). To prevent GVHD, patients are treated with immunosuppressive drugs, most commonly, calcineurin inhibitor combined with Methotrexate (5). Despite this, a majority of the patients developed acute GVHD, with a considerable mortality, even if this was significantly lower in children compared to adults (6). To confirm the gastrointestinal GVHD histopathological biopsies is recommended, since e.g., viruses could cause gastrointestinal symptoms (7–9). Cortisone is first-line therapy for acute GVHD (10) and almost all immunosuppressive therapies are used as a secondary treatment with varying degrees of success (11). Friedenstein et al. were the first to describe MSCs (12). We introduced mesenchymal stromal cells (MSCs) as a new therapy for acute GVHD (13, 14). MSCs are rare in all tissues in the body and can differentiate into several cells of mesenchymal cell lineages, such as bone, cartilage, tendon, cardiomyocytes, muscles, and fat (15, 16). There is no specific CD marker for MSCs. However, they stain positive for CD29, CD73, CD90, CD105, and CD166. They are negative for hematopoietic markers, CD34, CD45, and CD14. They are not true stem cells because they cannot regenerate and maintain a whole tissue compartment. MSCs express HLA class I molecules and contain intracellular HLA class II that is expressed on the cell surface after interferon-γ stimulation (17). After injection, MSCs do not appear to be long lived and have been demonstrated in the circulation only shortly after infusion into patients who underwent autologous HCT for breast cancer (18).

## Immunosuppression

MSCs have potent immunomodulatory effects and inhibit phytohemagglutinin induced T cell proliferation and alloreactivity in mixed lymphocyte cultures (MLC) (17, 19, 20). MSCs' inhibition of alloreactivity *in vitro* is independent of the major histocompatibility system (21). Furthermore, after differentiation into osteocytes, chondrocytes and adipocytes, immunosuppression was still induced (17). MSCs also prolonged skin allograft survival in baboons (19). Several factors and mechanisms are involved in MSC-mediated immune modulation.

Bone marrow MSCs (BM-MSCs) are susceptible to complement activation after contact with human blood (22). This results in cell dysfunction or cell death (23). When in contact with blood, BM-MSCs also elicit activation of clotting factors (24).

**Figure 1:**
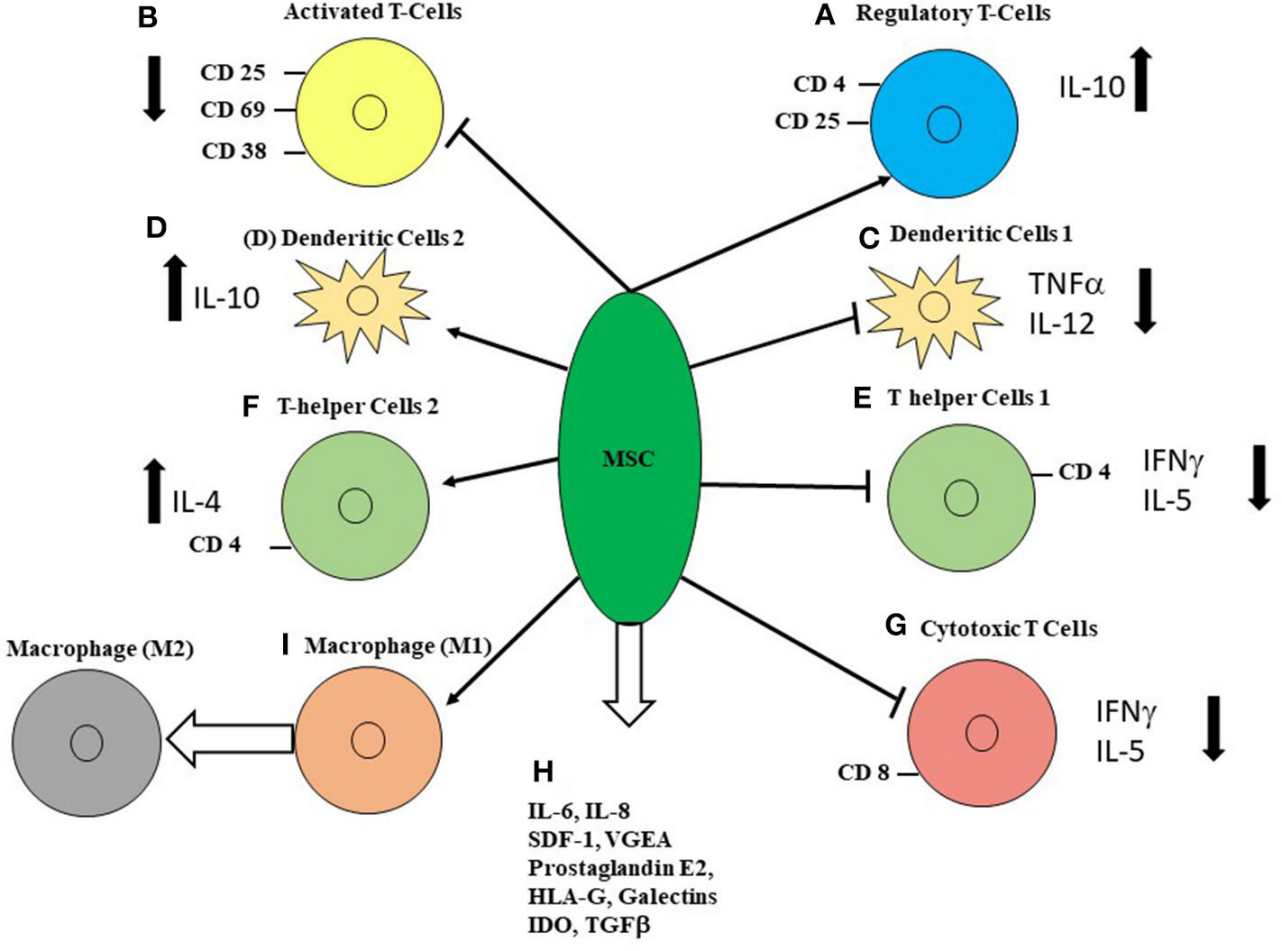
The multiple effects of MSCs on immune cells. **(A)** MSCs increase the proportion of CD4+CD25+ cells and IL-10 production. **(B)** MSCs decrease markers for activated T cells, CD25, CD69, and CD38. MSCs delayed maturation of APC and decreased expression of HLA-DR. **(C)** Dendritic cell type 1 when stimulated had decreased TNF-α and IL-12, when co-cultured with MSCs. **(D)** MSCs increased IL-10 secretion by LPS-stimulated dendritic cells type 2, CD4+ cell had decreased IL5-secretion. **(E)** T-helper cell type 1 IFN-γ production was significantly decreased by MSCs. **(F)** T-helper cell type 2 increased IL-4 secretion in the presence of MSCs. **(G)** MSCs inhibit mixed lymphocyte cultures and subsequent development of cytotoxic T cells by a soluble factor. **(H)** Several soluble factors are produced by MSCs, amongst them are IL-6, IL-8, stem-cell derived factor 1 (SDF1), vascular endothelial growth factor (VEGF). Soluble factors that have been suggested to inhibit T-cell activation are prostaglandin E2, which induces regulatory T-cells, indoleamine 2,3-dioxygenase (IDO), which is induced by IFN-γ which catalyzes the conversion from tryptophan to kynurenine and inhibits T-cell responses. Other soluble factors that have been suggested to inhibit T-cell responses are TGFβ1, hepatocyte growth factor and IL-2. **(I)** MSC induce macrophage differentiation from M1 to M2. (References are mentioned in the text).

MSC immunosuppression has been studied extensively (25–28). Stromal cells from various organs such as BM, Wharton's jelly, placenta tissues and cord blood have varying immunosuppressive effects in the MLC (17, 19–21, 29, 30). The MLC is also inhibited by skin fibroblasts (31). Immunosuppressive factors produced by MSCs include prostaglandin E2 (32), HLA-G5 (33), and galectins (34). MSCs also produce indoleamine-2,3, dioxygenase (IDO), which inhibits T cells by converting of tryptophan to kynurenine [(35), Figure 1]. IDO is involved in the induction of regulatory T cells and the inhibition of Th17 differentiation (36). IDO produced by MSCs also promotes differentiation of macrophages toward M2 phenotypes (37). MSCs also induce contact-dependent immunosuppression. Among these are activation of the PD-1 pathway (38), by activation of VCAM-1 and ICAM-1 (39), purification of CD39 and increased adenosine production (40), and Fas-mediated T-cell apoptosis (41). There are differences in various species and, in mice, several models failed to reduce alloreactivity and GVHD (42). To inhibit GVHD in mice, MSCs need to be licensed by IFN-γ, nitric oxide, or transduced with IL10 to prevent GVHD. In a colitis model in mice, it was shown that prevention of colitis by MSCs requires CD11b+ macrophages (43). In a murine model of GVHD, it was demonstrated that MSCs are actively induced to undergo perforin-dependent apoptosis by recipient cytotoxic T-cells, and that this process is essential to initiate MSC-induced immunosuppression (44). After IV infusion, recipient phagocytes engulf apoptotic MSCs and produce IDO, which is necessary for immune suppression. MSCs produce exosomes and microparticles, some of which are small complexed entities that contain both immunomodulatory proteins, micro RNA and mediators for homing abilities (45). Exosomes were also used to reverse acute GVHD (46).

## Mesenchymal Stromal Cells For Treatment of Acute GVHD

We introduced MSCs, as a therapy for acute GVHD, by treating a 9-year-old boy with life-threatening grade IV acute GVHD, as well as a phase-I study in GVHD patients whom were resistant to several immunosuppressive therapies (13, 14). We also performed a multi-center phase II study, including 55 patients with severe steroid resistant GVHD (47). Complete responders had lower transplantation-related mortality 1 year after infusion than patients with partial or no response (11 [37%] of 30 vs. 18 [72%] of 25; *p* = 0.002). Patients with complete response to MSCs had a 2-year survival of 53% as opposed to 16% in partial and non-responders. Children had a trend for better response (64%) as opposed to adults (47%). Subsequently, several single-center studies were performed with varying results using various sources of stromal cells, for instance, adipose tissue (48). Lucchini et al. gave platelet lysate expanded MSCs to children with severe steroid refractory acute or chronic GVHD with varying responses (49). Commercial MSCs (prochymal) were given to 12 children with therapy-resistant grade III and IV acute GVHD (50). A complete response was seen in seven children (58%), a partial response in two (17%), and mixed responses were recorded in three (25%) of the children. The 100-day survival was 58%. Osiris performed a double-blind placebo controlled phase 2/3 study using prochymal for severe acute GVHD (51). The children were given 8 × 10^6^ MSCs/kg twice a week or placebo. Among 260 patients, including children and adults, who were randomized in this trial, a complete response at 28 days was 74% in the MSCs group and 30% in the placebo group (52). However, the 180-day durable response of liver GVHD was 29% in the MSC group compared to 5% in the placebo group (*p* = 0.047%). Among patients with acute GVHD grades III–IV, Remestemcell-L demonstrated significantly higher overall response, 65%, as opposed to 23% in the placebo arm (*p* = 0.05). Children had a better outcome of treatment with MSCs for acute GVHD as compared to adults. These pediatric patients were also reported separately (53). Ball and coworkers reported on 37 children treated with MSCs for steroid-refractory grade III–IV acute GVHD (54). A response was observed in 65% of the children. The 3-year survival was 37%. Kurtzberg et al. reported on 241 children with steroid refractory acute GVHD who were treated for 4 weeks with infusion of 2 × 10^6^ MSCs/kg (Remestemcel-L) twice weekly (55). The overall response rate at day +28 was 65%. Survival at 100 days was 82% among the responders and 39% among the non-responders (*p* ≤ 0.001). In a Brazilian multicenter study, involving 16 children and 30 adults with steroid refractory GVHD, half of the patients responded and 1-year survival was 20% (56). A study using platelet-lysate-expanded MSC for steroid refractory acute GVHD included eight children and 22 adults. The overall response rate at day +28 was 50% in the adults and 88% in the children (*p* = 0.099). The survival was 88% in the children as opposed to 25% in the adults (*p* = 0.003) (57).

A study used BM-MSCs pooled from multiple third-party donors (58). The study included 92 adult and pediatric patients with steroid refractory acute GVHD. The patients received a median of three doses of pooled MSCs without toxicity. The overall response was 82% and 6-month survival was 64%. In a previous separate analysis of children, the overall response at day 28 was 77% and the 2-year survival was 77% (59). At our unit, long-term follow up of patients treated with BM-MSCs with steroid refractory GVHD included nine children and 22 adults (60). Two-year survival was only 26%. Patients receiving MSCs from passage 1–2 had significantly better survival than those receiving MSCs from passage 3–4 (*p* < 0.01). A meta-analysis reported that children had a better response to MSCs therapy for steroid refractory acute GVHD, with an overall response rate of 82%, as opposed to 70% in adults (*p* = 0.04) (61). A more recent meta-analysis included children and adults given MSCs for prophylaxis (*n* = 651) and for treatment of acute GVHD (*n* = 149) and chronic GVHD (*n* = 76) (62).

## Mesenchymal Stromal Cells For Treatment of Chronic Graft-Versus-Host Disease

Chronic GVHD is a great burden for many patients after HCT (63, 64). It seems logical to use MSCs to treat chronic GVHD, which resembles auto-immune disorders. MSCs were reported to be successful in many models of autoimmune diseases (65, 66). There are only a few reports on MSCs for chronic GVHD and most are about adults (14, 67, 68). Lucchini et al. used platelets-lysate expanded MSCs in four children with chronic GVHD (49). Transient benefits were noted. One child had a complete response that subsequently re-flared.

DSCs appear to have a stronger immunosuppressive effect than MSCs from bone marrow (30, 69). Thus, we used DSCs to treat chronic GVHD in three pediatric patients with severe grade 3 chronic GVHD (Based on National Institute of Health, NIH) (70). The three pediatric patients were affected in several organs such as the skin, mouth, eyes, gastrointestinal tract, liver, lungs and joints, fascia. Two patients received two doses of DSC and one patient received one dose. Two patients had a partial response in the liver, normalization of elevated liver enzymes and, in one patient, esophageal varices disappeared. However, the overall grading of chronic GVHD remained very severe (3) according to NIH grading (71). A meta-analysis of 76 children and adults with chronic GVHD suggested improved survival using MSCs (62).

## Prevention of GVHD and Graft Failure

In mice, MSCs were shown to prevent the development of lethal GVHD (72). Lazarus et al. performed co-transplantation of HLA-identical sibling bone marrow and donor MSCs in 46 patients (73). No patient had graft failure and grades III–IV acute GVHD were seen in 15% of the patients. We performed co-transplantation of HCT and MSCs to enhance engraftment (74). All patients had engraftment and full donor chimerism. A prospective randomized study of HCT and with co-infusion of MSCs or placebo reported decreased risk of acute GVHD and increased likelihood of relapse (75). Engraftment of neutrophils and platelets was similar in the two groups. Most studies of co-transplantation of HCT and MSCs are performed in adult patients or in a combination of pediatric and adult patients (76, 77). In a pediatric study, parental haplo-identical MSCs were used to promote engraftment in unrelated donor umbilical cord blood transplantation (78). In another pediatric study, MSCs were given to recipients of haplo-identical grafts (79). No patient had graft failure as opposed to 10% of the retrospective controls. A meta-analysis, which included 651 children and adults, showed improved survival in patients treated with MSCs as prophylaxis (62). MSCs may also be used to treat graft failure (80, 81).

## MSCs For Metabolic Disorders

Hurler's disease is deficiency of the enzyme alfa-L-iduronidase. HCT may partially prevent disease progression if performed before the patient is 2 years of age (82, 83). HCT patients with Hurler's disease and metachromatic leukodystrophy were given MSCs to enhance enzyme production after HCT (84). The rationale for using MSCs was because these cells express high levels of alpha-L-iduromidase and arylsulphatase-A. Four out of five patients with metachromatic leukodystrophy had improved nerve conduction velocity. Five patients with osteogenesis imperfecta who underwent HCT had donor osteoblast engraftment, new dense bone, increased total bone mineral content and improved growth velocity (85). The frequency of bone fractures decreased. Gene-marked MSCs were given to six HCT patients with MSC engraftment in bone and accelerated growth velocity. In a fetus with bilateral femur fractures due to severe osteogenesis imperfecta, *in utero* transplantation of MSCs showed 7% engraftment and the patient had fewer fractures than expected after birth (86).

## MSCs For Hemorrhages and Side-Effects

We used MSCs for hemorrhagic cystitis, colon perforation, and pneumomediastinum after HCT (87). Adult patients are more vulnerable and had more toxicity after HCT as opposed to pediatric patients. However, toxicity also occurs in children with advanced hematological malignancies treated with multiple rounds of chemotherapy prior to transplantation. Stromal cells induce clotting and may stop or prevent bleeding. This effect appears to be stronger for DSCs than BM-MSCs (88). Yim et al. reported on two patients with pneumomediastinum/pneumothorax with resolution after MSCs treatment (89).

## Materials and Methods

### Patients

Six children diagnosed with grade II–IV acute gastrointestinal GVHD, with or without skin involvement, were treated with DSCs (Table 1). The patients comprised five boys and one girl aged from 10 months to l6 years. Informed consent was obtained from the legal guardians of the patients. Diagnoses were pre-B-ALL in two children, Langerhans cell histiocytosis (LCH), sickle cell anemia, osteopetrosis, and severe combined immunodeficiency (SCID). The conditioning therapy was total body irradiation and etoposide in the two patients with leukemia. The four children with other disorders were given fludarabine together with treosulfan in three patients and with the addition of thiotepa in one patient with sickle cell anemia. A boy with osteopetrosis was given a low dose of busulfan, in addition to fludarabine. Donors were matched unrelated in three patients, cord blood in two children, and bone marrow from an HLA-identical sibling donor in one patient. Post-transplant immunosuppression consisted of tacrolimus together with sirolimus in four patients (Table 1). Three patients were given antithymocyte globulin (90).

**Table 1:** Characteristics of pediatric patients treated with DSCs for acute GVHD.

**No**	**UPN**	**Sex/age**	**Diagnosis**	**Conditioning**	**Donor**	**Graft**	**Immuno-suppression**	**Acute GVHD grade**	**Organs involved**	**Day of acute GVHD**	**Day of DSC**
1	1555	M/1	Langerhans cell, histiocytosis	Flu treo	UD	CB	Prograf rapamune	III SR	GI skin	+ 20	+23, +43
2	1625	F/16	High risk pre B-ALL	TBI+VP16	MUD	BM	Prograf rapamune ATG	II SR	GI skin	+ 18	+30
3	1687	M/9	Intermediate risk pre B-ALL	TBI+VP16	HLA id sister	BM	Prograf rapamune	III SR	GI skin	+ 9	+31, +38, +45, +55, +284, +298
4	1692	M/14	Sickle cell anemia	Flu treo TT	MUD	BM	Prograf rapamune ATG	II SR	GI	+ 182	+200
5	1707	M/1	Osteopetrosis	Flu Bu2	MUD	PB	Prograf methotrexate ATG	II SR	GI	+ 17	+ 21, +70, +78, + 86, +93
6	UAH	M/1	SCID	Flu treo	UD	CB	Cyclosporine MMF	IV SR	GI skin	+ 33	+ 57, +64, +71, +78, +92, +113

*M, male; F, female; Flu, Fludarabine; Treo, Treosulphan; UD, unrelated donor; CB, cord blood; GI, gastro-intestinal tract; TBI, total body irradiation; VP16, vepesid; BM, bone marrow; ATG, anti-thymocyte globulin; TT, Thiotepa; Bu, short course busulphan; SR, steroid refractory; MUD, matched unrelated donor; HLA id, human leukocyte antigen identical; UAH, Uppsala Academic Hospital; SCID, severe combined immunodeficiency; MMF, murophenolate mofetil*.

Acute GVHD was graded according to Seattle criteria (91). The diagnosis of gastrointestinal GVHD was based on biopsies from endoscopies (7–9). Skin biopsies were not performed. Donor recipient chimerism was followed by PCR and patients with acute GVHD were full-donor chimeras (9, 92). Cytomegalovirus (CMV) was followed weekly by PCR and reactivation was treated with ganciclovir (93). Epstein-Barr virus (EBV) PCR was only regularly followed in patients with an EBV-mismatched donor (94). Adenovirus was not monitored routinely (95), and only when an infection was suspected.

### Ethics

We received ethical approval from the regional ethic committee to harvest DSCs from Caesarian section placentas and use them for treatment of GVHD and toxicity after HCT (2009/418-31-34 and 2010/2061-32, 2010/452-31/4, and 2014-2132-32). The procedure for using DSCs was also later approved by the Central Ethical Review Board in Sweden (Dnr 011-2016). The method for clinical culture of DSCs was also approved by the Swedish Product Agency (Dnr 6.1.3-42994/2013).

### Decidua Stromal Cell Culture

The method to culture and expand DSCs was previously published in detail (96). DSCs express CD166, CD105, CD73, CD44, and CD29. They did not express hematopoietic markers CD34, CD14, and CD45. DSCs were negative for bacteria, mycoplasma, and fungi before infusion. The DSCs were cultured and expanded in a good manufacturing process laboratory. DSCs were stored in liquid nitrogen, thawed, and resuspended in CliniMACS PBS/EDTA buffer, supplemented with 10% AB plasma or 5% albumin (69). The cells were washed three times and resuspended in NaCl and 10% AB serum or 5% albumin. The infusion solution was filtered through a 70 μM cell strainer (BD Bioscience, Franklin Lakes, NI) before being transferred to a heparinized syringe (Leo Pharma, Ballerup, Denmark) at 2 × 10^6^ cells/ml. The DSC was infused intravenously using a central venous line. The central venous line was flushed with 2–5 mL of NaCl with 25 IE heparin/ml in children weighing over 15 kg and 12.5 IE heparin/ml in children weighing under 15 kg.

## Results

**Patient 1** (UPN, unique patient number, 1555). A male baby boy was presented with disseminated LCH disease including bone marrow involvement and was pretreated with steroids and chemotherapy, followed by a HCT. The boy received an unrelated cord blood transplant. We previously reported that LCH can be cured by HCT (97, 98). Due to poor engraftment he was treated with granulocytes colony-stimulating factor (G-CSF) from day +20 after HCT. He reached absolute neutrophil counts (ANC) >0.5 × 10^9^/L on day +27. On day +20 after HCT, he started vomiting and had watery diarrhea 10 times/day. His diarrhea deteriorated and he developed a skin rash on the back of his body. He was given high- dose prednisolone (2 mg/kg). Due to unresponsiveness he was treated with DSCs 3 days later and one additional dose was administered 3 weeks after the steroids had been introduced (Table 1). DSC doses were above 2 × 10^6^/kg and viability was 78 and 95% in the two infusions, respectively (Table 2). At day 28 after DSC infusion, he had a partial response (PR). At day 56 and at the 6-month follow-up he showed no signs of acute GVHD (Table 2). He was diagnosed with a CMV reactivation on day +61 treated with ganciclovir. He is currently alive and well more than 8 years after HCT and from last follow up he showed Lansky score of 100%.

**Table 2:** Decidua stromal cells (DSCs) for therapy of acute GVHD characteristics, cell dose and outcome.

**Patient**	**UPN**	**DSC dose no**	**DSC viability %**	**Cell dose × 10^**6**^/kg**	**Passage**	**Day 28 response**	**Day 56 response**	**6 months response**	**Chronic GVHD grade**	**Outcome**
1	1555	1	78	2.7	2	PR	CR	CR	0	Alive and well, Lansky score 100%
		2	95	2.4	2					
2	1625	1	91	1.7	3	CR	CR	CR	0	Died from leukemic relapse 2 years post HCT
3	1687	1	97	1.2	4	CR	PR	PR	2	Moderate chronic GVHD obstructive bronchiolitis. Presently Lansky score 100%
		2	69	1.1	3					
		3	96	1.1	4					
		4	96	1.1	4					
		5	96	1.2	4					
		6	94	1.5	3					
4	1692	1	96	0.9	4	PR	PR	PR	2	Died from cerebral hemorrhage
5	1707	1	89	1.9	4	CR	PR	CR	0	Alive and well. Lansky score 100%
		2	100	1.7	4					
		3	91	1.5	4					
		4	95	1.6	4					
		5	82	1.5	3					
6	UAH	1–6	69–100	1.0	3–4	PR	CR	CR	0	Alive and well, Lansky score 100%

*PR, partial response; CR, complete response; UAH, Uppsala Akademic Hospital*.

**Patient 2** (UPN 1625). A 16-year-old female with high risk B-ALL in 2nd complete remission (CR) received bone marrow from an unrelated donor. The patient was treated pre-HCT according to the NOPHO (Nordic Pediatric Hematology Oncology) ALL protocol 2008 and was in complete remission pre HCT, including MRD <0.01% (99). She experienced CMV reactivation on day +19, treated with ganciclovir. ANC reached >0.5 × 10^9^/L on day +23. Eighteen days post-transplant, she developed steroid refractory grade II acute GVHD of the gastro-intestinal tract and a skin rash. She was treated with a high dose of steroids from day +20, but did not respond. Due to steroid resistance, she was treated with one dose of DSCs 30 days after HCT (Table 1). The DSC dose was 1.7 × 10^6^/kg with 91% viability (Table 2). Her symptoms of acute GVHD disappeared and she was considered to be in a complete response at day 28 and remained so. However, the patient died from leukemic relapse 2 years after HCT.

**Patient 3** (UPN 1687). A 9-year-old boy with an intermediate risk of B-ALL in CR2 received a bone marrow graft from his HLA-identical sister. He was previously treated according to the NOPHO ALL protocol 2008 (99). Both donor and recipient were CMV seropositive. He had no CMV reactivation. On day +10 he had hemorrhagic cystitis grade II that resolved. Already on day 9 after HCT he developed acute GVHD of the gastrointestinal tract and erythema of the skin. He did not respond to high doses of steroids and was considered steroid refractory. On day +30 he also developed a varicella-zoster reactivation. One month after HCT he was given 1.2 × 10^6^ DSC/ × 10^6^/kg with a viability of 97% (Table 2). At day 28 after DSC treatment was initiated, he had complete resolution of all signs of acute GVHD but received another three additional weekly doses (Tables 1, 2). However, at 6 months, it was evident that he had developed signs of chronic GVHD as sicca and lichenoid changes of the skin, treated with extracorporeal psoralene and ultraviolet light (PUVA). After another 2 months he developed signs of a more generalized GVHD, with symptoms from both the skin, the liver, and the gastrointestinal tract. The biopsy from the GI-tract revealed GVHD, grade II (8) and he was given two more doses of DSC (Tables 1, 2). The symptoms of acute GI-GVHD disappeared but one and a half year after transplant he was still having symptoms of moderate chronic GVHD, mainly symptoms of bronchiolitis obliterans. 6.5 years after HCT he is suffering from NIH grade 2 chronic GVHD and is now treated with a JAK2 inhibitor, but from his last follow up he scored Lansky 100%.

**Patient 4** (UPN 1692). A 14-year-old boy arrived in Sweden, from an African country with an untreated severe sickle cell disease. He had a history of multiple sickle cell crises, as severe pain, osteonecrosis, cerebral infarctions, and bleedings and was therefore planned for a HCT. Before HCT he was treated with Hydrea capsules, but the treatment showed very moderate effect. He was finally transplanted and received bone marrow (0.25 × 10^6^ CD34+ cells/kg) from an unrelated donor (12/12 match). He reached ANC >0.5 × 10^9^/kg on day +19. On day +28 he was treated with acyclovir for a herpes simplex virus infection. Immunosuppression was tacrolimus combined with sirolimus. During discontinuation of immunosuppression on day 182 after HCT he developed diarrhea diagnosed as gastrointestinal GVHD. Steroids were administered, but the diarrhea continued. One week later he was given 0.9 × 10^6^ DSC/ × 10^6^/kg (Table 2). He had a partial response at 28 days and at follow-up at 6 months. Seven months after HCT he had CMV reactivation treated with ganciclovir. One year after the transplant he developed chronic GVHD, NIH overall score 2 (Table 2). However, the patient died from severe cerebral hemorrhage 1 year and 9 months after HCT, where previous cerebral damage pre HCT probably contributed to cerebral hemorrhage post HCT.

**Patient 5** (UPN 1707). A 1-year-old boy with osteopetrosis rejected the first graft and was re-transplanted 2 months later. He received a peripheral blood graft from an unrelated donor (Table 1). HLA-match was 10/12 with one antigen-HLA-C and –DP-mismatches. CD34+ cell dose was 34 × 10^6^/kg. He had CMV reactivation on day +11, treated with ganciclovir. On day 17 after HCT he developed diarrhea grade II that did not respond to steroids. He was subsequently given five doses of DSC in doses ranging from 1.5 to 1.9 × 10^6^/kg per kg (Table 2). The viability of the cells ranged from 82 to 100%. At day 28 after initiation of DSC therapy, he had a complete response. At day 56 he had some abdominal pain and a loose stool. At the 6-month follow-up the stool was normal. He did not develop any chronic GVHD and is currently alive and well 6 years after transplantation, with a Lanskys score of 100%.

**Patient 6** The boy, born at term, non-consanguineous parents, was admitted to the hospital at the age of 9 months, with symptoms of severe respiratory infections, failure to thrive, and low lymphocytes. He was investigated for suspected severe combined immunodeficiency (SCID). Genetic analysis revealed a JAK-3 gene mutation (two heterozygous variants, leading to a frame shift and premature stop codon; p.Ser 449LysfsX71). At 12 months of age the boy was transplanted, with cord blood as a stem cell source. Pre-HCT the boy was colonized with rhinovirus, which also was observed after transplantation. On day 29, PCR-chimerism analysis revealed 60% donor cells. Subsequently, during immunosuppressive tapering, he developed a skin rash and, a few days later, also massive diarrhea due to gastrointestinal GVHD. This was diagnosed on a colon biopsy showing crypt destruction with several apoptotic bodies and regenerated features of grade IV gastrointestinal GVHD (8). He did not respond to steroids or mycophenolate mofetil therapy (Tables 1, 2). From day 57 after HCT, he was treated with weekly doses of DSCs. He had a partial response at day 28 and continued to need albumin transfusions. He received a total of six doses of DSCs before the resolution of gastrointestinal GVHD. At day 56 and 6 months after transplant he had a complete response and was doing well. Apart from rhinovirus, no viral, or fungal infections were diagnosed post-HCT. He is currently alive and well, 5 years after transplantation. He doesn't need any medications goes to school and shows Lanskys score of 100%.

### Overall Follow Up

The outcome among these six children treated for severe gastrointestinal and sometimes also acute skin GVHD at the 28-day follow-up was a complete response in three patients and a partial response in three patients (Table 2). At 6 months, a complete response was seen in four patients and a partial response in two patients. Two patients developed moderate chronic GVHD. One patient with high risk pre-B-ALL died of leukemic relapse 2 years after transplantation. A boy with sickle cell anemia died of cerebral hemorrhage 1 year and 1 month after HCT, although he had a history of multiple severe sickle cell crises before HCT. Three patients are alive and well and one patient is suffering from moderate chronic GVHD with obstructive bronchiolitis but responded to Jak-2 inhibition. Now he scores Lansky 100%. Overall, there is a 5-year survival of 67%.

## Discussion

Although this is only a small series of pediatric patients treated for acute GVHD, it still holds some promise. None of the children died from GVHD and 6-year survival was four out of six (67%). This is similar to what was achieved with DCSs with 21 patients, most of them older adults with a 4-year survival of 57% (100). The two deaths were due to relapse in the patient with high-risk ALL and cerebral hemorrhage in the patient with sickle cell disease. These are unfortunate yet expected complications after HCT. Patients who survived acute GVHD have an reduced risk of leukemic relapse (101). The graft-versus-leukemia (GVL) effect did not prevent relapse in this girl with high risk B-ALL. She did not develop chronic GVHD. The study from the International Registry suggested that acute GVHD had a profound GVL effect in ALL patients (102). A European study in ALL patients found that chronic GVHD was more important to decrease relapse probability (103). There were only two patients who developed moderate chronic GVHD. Children have a relatively low risk of chronic GVHD (104, 105). However, there is an increased risk of chronic GVHD in patients who survive acute GVHD (101). Children have a better outcome than adults after HCT and this is striking in patients with severe acute GVHD (6). In a prospective randomized study performed by Osiris, it was reported that children treated for severe acute GVHD, as opposed to adults, had a better outcome (51). The first multicenter study using MSCs for acute GVHD also showed a better outcome in children than adults (47). However, this was not supported by a meta-analysis, which showed that survival following MSC therapy for acute GVHD did not differ in children and adults (106).

An advantage of using MSCs as opposed to other drugs to treat acute GVHD is safety, with few, if any side-effects (107, 108). There were no side-effects caused by the stromal cells in any of the six children treated with DSCs.

The ideal source of stromal cell for treatment of acute GVHD, MSC from bone marrow, adipose tissue, Wharton's jelly, umbilical cord, placenta tissue or DSCs, may be discussed. In a humanized mouse model, it was shown that MSCs from BM, umbilical cord, and adipose tissue had different properties (109). In Table 3 is listed the different properties of MSCs from bone marrow compared to DSCs. Bone marrow aspiration is quite a painful procedure. Thus, alternative sources such as adipose tissue, cord, placenta tissue, or fetal membrane, stromal cells are more easily accessible. We found that DSCs had a stronger immunosuppression of alloreactivity *in vitro* in mixed lymphocyte cultures compared to MSCs from other sources. We therefore selected DSCs for further investigation (30). DSCs also appeared to be more effective for treating acute GVHD compared to BM-MSCs (69). However, it is unlikely that different sources of stromal cells will be compared in prospective randomized studies for the treatment of acute GVHD. Currently, there are several promising drugs for treating acute GVHD, such as Ruxolitinib, Vedolizumab and Etanercept (110–112). However, it seems that an advantage of using MSCs is the toxicity profile.

**Table 3:** Differences between bone marrow-derived mesenchymal stromal cells and placenta-derived decidual stromal cells.

**Characteristic**	**MSC**	**DSC**
Expansion potential	++	++++
Differentiation to fat and cartilage	+++	+/–
Size, volume	4600 fl	2400 fl
Express PDL-1, PDL-2	+	++
Express CD49d, homing to inflammatory tissue (integrin)	+	++
Vascular cell adhesion molecule 1 (VCAM-1) expression	+	–
Express HLA class II after IFNγ stimulation	+	–
Pro-coagulant tissue factor	6%	39%
CD55 complement regulatory activity	62%	98%
Reduction in clotting time	55%	70%
Prevent alloreactivity *in vitro* (MLC)	++	+++
Needs direct contact for immunosuppression	–	+
Overall response in steroid refractory acute GVHD	75%	100%

*MLC, mixed lymphocyte culture*.

The first child (UPN1555) was treated with G-CSF for poor engraftment. G-CSF was reported to be associated with severe acute GVHD because it can trigger alloreactive T-cells (113, 114). G-CSF may have potentiated acute GVHD in this child.

Several large studies have been using MSCs, as shown from a single report on children from Kurtzberg et al. who recently reported on 241 children with grade II–IV steroid refractory acute GVHD (115). The 28-day overall response rate was 65% with a 14% complete response. The 100-day survival was 67%. These results were achieved with the commercially available MSCs Remestemcel-L. The randomized study by Osiris, which did not show an overall improvement in the placebo-controlled trial, showed that pediatric patients had a significantly better outcome using MSCs compared to the placebo group (53). Bonig et al. used MSCs pooled from multiple donors to treat acute GVHD (58). They reported an overall response rate of 82% following a median of three doses of pooled MSCs. Overall, 6-month survival was 64%.

MSCs have mainly been used for treatment of acute GVHD in pediatric patients. They have not been used much for chronic GVHD. This is because stromal cells have a strong anti-inflammatory effect, which may be more effective for acute inflammatory processes such as acute GVHD and less effective in chronic fibrotic processes (116). Another indication for MSCs, mainly used in adults, is hemorrhagic cystitis (117, 118). MSCs have also been used for the treatment of acute respiratory distress syndrome (ARDS). There is a wealth of experimental data suggesting the potential of MSCs for sepsis and ARDS (119–121). We treated a young boy who developed ARDS after HCT with MSCs (122). He died from massive Aspergillus infection. DSCs were shown to dramatically reverse ARDS in a male patient early after HCT (123). There is limited clinical experience (124). The lack of data on pediatric patients for these more novel indications could be because they are under development. If effective in the adult studies, MSCs will also be used for hemorrhagic cystitis, ARDS, and other indications that are more experimental today.

In addition to acute GVHD, MSCs have also been used to prevent and reverse graft failure, enhance engraftment, or as prophylaxis to reduce GVHD (74, 79–81). These studies include pediatric patients and adults.

As discussed above, the immunosuppressive effects of MSCs are induced by direct contact, as well as via several soluble factors. Exosomes and microvesicles derived from MSCs were shown to protect from acute kidney injury (125), myocardial ischemia (126), and pulmonary hypotension (127) in animal models. Exosomes for MSCs were also demonstrated to reverse severe acute GVHD (46). Since exosomes will only transfer soluble effect by MSCs and not a direct immunosuppressive effect, it is less likely that exosomes will replace MSCs in the near future.

To conclude, MSCs from various sources are mainly used in pediatric patients to treat severe acute GVHD and have shown encouraging response rates and survival efficacy. Thus, commercially available MSCs are registered as a drug in Canada, Japan and New Zeeland (128). Furthermore, MSCs also have the potential to cure other acute inflammatory and toxic disorders seen in pediatric patients, such as hemorrhages, ARDS, poor engraftment, and possibly also neuroinflammatory disorders (129).

## Data Availability Statement

The datasets presented in this article are not readily available because There is no restriction for the authors of this article to use this datasets. However it is not publicly available. The reviewers are of course welcome to request any data they feel are of importance for evaluating the study properly. Requests to access the datasets should be directed to Olle.ringden@ki.se; Britt.Gustafsson@ki.se.

## Ethics Statement

The studies involving human participants were reviewed and approved by Ethical committee of Karolinska Institutet, Stockholm, Sweden. Approval to collect placentas and isolate decidual stromal cells (DSC), Dnr 20019/413-31/4 and 2010/2061-32. To use DSCs for treatment of acute GVHD and toxicity after hematopoietic cell transplantation (Dnr 2010/452-31/4 and 2014/2132-32). Written informed consent to participate in this study was provided by the participants' legal guardian/next of kin.

## Author Contributions

OR, BG, and BS: concept and design, collection and assembly of data, manuscript writing, and final approval of manuscript. OR and BG: financial support. OR and BS: administrative support. All authors contributed to the article and approved the submitted version.

## Conflict of Interest

The authors declare that the research was conducted in the absence of any commercial or financial relationships that could be construed as a potential conflict of interest.
